# Therapeutic potential of ADAM10 modulation in Alzheimer’s disease: a review of the current evidence

**DOI:** 10.1186/s12964-023-01072-w

**Published:** 2023-03-14

**Authors:** Mohammad Rafi Khezri, Mehdi Mohebalizadeh, Morteza Ghasemnejad-Berenji

**Affiliations:** 1 Student Research Committee, Urmia University of Medical Sciences, Sero Road, Urmia, 5715799313 Iran; 2 Systematic Review and Meta-Analysis Expert Group (SRMEG), Universal Scientific Education and Research Network (USERN), Tehran, Iran; 3 Department of Pharmacology and Toxicology, Faculty of Pharmacy, Urmia University of Medical Sciences, Urmia, Iran; 4 Research Center for Experimental and Applied Pharmaceutical Sciences, Urmia University of Medical Sciences, Urmia, Iran

**Keywords:** ADAM10, Alzheimer’s disease, Amyloid β, Natural compounds

## Abstract

Alzheimer’s disease (AD), the most common neurodegenerative disease worldwide, is caused by loss of neurons and synapses in central nervous system. Several causes for neuronal death in AD have been introduced, the most important of which are extracellular amyloid β (Aβ) accumulation and aggregated tau proteins. Increasing evidence suggest that targeting the process of Aβ production to reduce its deposition can serve as a therapeutic option for AD management. In this regard, therapeutic interventions shown that a disintegrin and metalloproteinase domain-containing protein (ADAM) 10, involved in non-amyloidogenic pathway of amyloid precursor protein processing, is known to be a suitable candidate. Therefore, this review aims to examine the molecular properties of ADAM10, its role in AD, and introduce it as a therapeutic target to reduce the progression of the disease.

**Video abstract**

**Supplementary Information:**

The online version contains supplementary material available at 10.1186/s12964-023-01072-w.

## Background

Alzheimer’s disease (AD) is known as the most common chronic neurodegenerative disease. As the disease progresses, it disrupts the patient's memory and cognitive functions [1]. AD is characterized by aggregation of amyloid β (Aβ)-containing extracellular plaques and tau-containing intracellular neurofibrillary tangles in the neurons [2, 3]. However, AD initially presents with transient forgetness, but as the disease progresses, other symptoms such as impaired speech and vision and long-term memory dysfunction [4]. Although the rate of progression can vary, the average life expectancy after diagnosis is three to nine years [5].

AD is a multifactorial disease, which means environmental factors also play an essential role in addition to genetic factors. However, only up to 2% of AD cases are inherited, known to start early and progress faster [6]. In sporadic AD, genes encoding amyloid precursor protein (APP), PSEN1, and PSEN2 are known to be critical factors, which are involved in over-production of Aβ [7, 8], which is a significant component of Aβ plaques. Two other mutations in the ABCA7 and SORL1 genes have recently been observed in patients with the familial AD [9].

Most AD cases are not inherited and experience the onset of symptoms at an older age, typically 65 years. More than 600 genes have been studied as AD susceptibility agents. The most potent genetic risk factor for disseminated AD is APOEε4 [10], one of the four alleles of Apolipoprotein E. Between 40 and 80% of people with AD have at least one APOEε4 allele [11]. The APOEε4 allele triples the disease risk in heterozygotes and 15-fold in homozygotes. Some studies have also shown that alleles in the TREM2 gene are 3–5 times more likely to develop AD [12].

It is not yet clear how the production and accumulation of Aβ cause pathogenesis. The amyloid hypothesis refers to the accumulation of Aβ peptides as the main event that causes neuronal destruction. Accumulation of Aβ fibrils, believed to be responsible for impaired cell ion homeostasis, leading to induce neuronal apoptosis. It is also known that Aβ is selectively present in mitochondria in brain cells. It is made with AD and also inhibits the functions of certain enzymes and the use of glucose by neurons [13, 14].

Despite many efforts to understand the pathophysiology of AD, no definitive cure has been identified yet. Increasing evidence suggest that designing treatment regimens to target the factors involved in the pathophysiology of the disease can be constructive. One of the best treatment candidates for AD appears to be the Aβ production pathway, where a variety of enzymes and intracellular factors are involved. In this regard, numerous studies introduce that inducing the non-amyloidogenic APP processing pathway and inhibition of amyloidogenic APP processing can be an effective therapy in AD. A disintegrin and metalloproteinase domain-containing protein (ADAM) 10 is one of the most important proteases involved in APP processing, which is shown its activation leads to reduce Aβ production and exhibits a protective role agains AD. Therefore, this review focuses on the role of ADAM10 in pathophysiology of AD, and introduces it as a probable therapeutic target to reduce disease progression.

### ADAM10 structure and synthesis

ADAM is a family of metalloproteinases consist of approximately 750 amino acids with proteolytic activity to process ectodomain of diverse cell-surface receptors [15]. One of the most important members of this family is ADAM10 which is mostly known due to its role in the processing of the amyloid precursor protein (APP) [16]. However, ADAM10 is expressed in different cells, the most important of which are vascular cells, leukocytes neurons, and tumor cells [17]. ADAM10 is synthesized co-translationally via the rough endoplasmic reticulum, maturated and transported by the Golgi apparatus [18]. Removal of the ADAM10 pro-domain is the main change during its maturation which keeps ADAM10 in an inactive state through a cysteine switch mechanism in a way that coordinates the zinc ion in the catalytic site and prevents ADAM10 proteolytic activity [18]. Pro-protein convertase is involved in ADAM10 maturation through its cleavage in several sites such as PC7 in Golgi apparatus [19]. The pro-domain is required as an intramolecular chaperon for folding correction, and it seems not to has a mere inhibitory function in ADAM10 [19]. This process has been proved followed by finding a large proportion of ADAM10 in the Golgi apparatus in breast carcinoma cells by confocal microscopy [20]. In addition to pro-domain removal, N-glycosylation of ADAM10 at four positions occurs during its transport to the membrane [19]. The other main domains of ADAM10 structure include disintegrin and an inactive zymogen containing C-terminal [18]. Although disintegrin domain seems not to be necessary for ADAM10 protease activity [21], the short intracellular C-terminus appears to play a crucial role as it has been demonstrated that the cleavage of epidermal growth factor is impaired in ADAM10^−/−^ cells with overexpressed cytoplasmic domain deletion mutant of the proteinase [22]. Additionally, several binding sites have been noted for cytoplasmic domain of ADAM10 which seems to be involved in regulatory events, including two proline-rich putative Src homology 3 (SH3) binding domains [23] and a binding site for calmodulin [22]. The SH3 binding domains direct ADAM10 are involved in direct ADAM10 to the postsynaptic membrane in neurons, while juxtamembrane binding site is involved in ADAM10 basolateral localization in epithelial cells [24]. In addition to bio-synthesis of ADAM10, its translocation to the membrane is a process which has been considered in different studies to modulate its physiologic function. There are a series of intracellular factors involved in translocation of ADAM10 in different steps of its maturation. In this regard, synapse-associated protein-97 (SAP97) is known as one of the main factors which governs ADAM10 transport from the Golgi outposts to the synapse, without any effects on ADAM10 trafficking from the endoplasmic reticulum. Mechanistically, protein kinase C (PKC) has been shown to mediate phosphorylation of SAP97 SRC homology 3 domain which regulates SAP97 association with ADAM10 and its translocation from Golgi to synapse [25]. Due to the sufficiency of SAP97 in ADAM10 exit from the endoplasmic reticulum, further studies conducted to introduce several other factors involved in this process. In this case, a subgroup of tetraspanins consist of eight cysteines in the large extracellular domain (Tspan10, Tspan5, Tspan15, Tspan14, Tspan17 and Tspan33) were known to be involved in ADAM10 exit from the endoplasmic reticulum [26]. In addition, it has been reported that an arginine-rich (^723^RRR) sequence is responsible for ADAM10 retention in the endoplasmic reticulum and its inefficient surface trafficking [27].

The mature form of ADAM10 has a molecular weight of ∼ 65 kDa [19]. ADAM10 ectodomain shedding leads to leave a membrane-anchored C-terminal fragment with a ∼ 10 kDa of weight and release of a ∼ 55 kDa soluble ADAM10. This process shows that regardless of the protease activity of ADAM10, this factor itself is affected by other proteases the most important of which are ADAM9 and 15 and γ-secretase [28]. One of the main components of γ-secretase, presenilin, affects ADAM10 leading to release its intracellular domain. This released domain is translocated to nucleus which thought to play a part in gene regulation [28].

### ADAM10 as a biomarker in Alzheimer’s disease

ADAM10 has previously been present in human CSF in several forms: an immature form that retains protamine, an unprocessed form, and a large cut solution form [29]. However, studies in AD patients have shown that the expression of ADAM10 in their platelets is associated with changes. Initially, there were reports of a decrease in ADAM10, but further studies reveal no significant link between ADAM10 levels and cognitive symptoms in AD patients, so it has been proposed that these changes might be due to the medications taken by patients [30]. Manzini et al. in a study examined ADAM10 levels in AD patients compared with healthy individuals who have reported increased levels of its substrates in patients' platelets (17).

This evidence suggests that ADAM10 might be used as a biomarker for AD diagnosis, although further research is needed to corroborate this theory. Table 1 summarizes the studies indicating ADAM10 alterations in samples from AD patients.

**Table 1:** Dysregulated ADAM10 in samples from AD patients

Author	Year	Country	Specimen	Findings	References
Aitana Sogorb-Esteve et al.	2018	Spain	CSF	proADAM10 levels remained unaltered decrease in ADAM10f and sADAM10	[29]
Izabela Pereira Vatanabe et al	2021	Brazil	Plasma and CSF	Increased plasma and CSF ADAM10	[99]
Maria Patrícia A. Oliveira Monteiro et al.	2021	Brazil	Plasma	Increased plasma ADAM10	[100]
Colciaghi et al.	2004	Italy	Platelet	Decreased Platelet ADAM10	[101]
Patricia Regina Manzine et al	2015	Brazil	Platelet	Decreased Platelet ADAM10	[102]
Anna Di Maio et al.	2022	Italy	Post-mortem brain tissue	Unchanged	[103]
Lynn M. Bekris et al.	2012	USA	CSF	Decreased ADAM10	[104]
Wen-Hui Huang et al.	2018	China	Plasma	Decreased ADAM10	[105]
Minji Kim et al.	2009	USA	Plasma	Increased ADAM10	[106]

## Roles of ADAM10 in Alzheimer’s disease

### ADAM10 and Aβ in Alzheimer’s disease

The most known activity of ADAM10, as a main α-secretase enzyme [31, 32], is its role in processing the APP. APP, a type I transmembrane glycoprotein, is expressed in different mammal cells, especially neurons. APP is known because it serves as Aβ precursor, including 12–15 residues of the membrane-spanning and 28 amino acids of the extracellular region of APP [33]. Although the underlying cause of AD remains unknown, Aβ accumulation as plaques is known to be a hallmark of the disease because of its association with the other processes involved in AD pathophysiology, such as oxidative stress and neuroinflammation [34]. Due to this issue, in recent years, therapeutic interventions to slow the progression of AD have been focused on reducing Aβ production. In the processing of APP, there are two pathways which addressing them can help understand the role of ADAM10 in AD pathophysiology. In Amyloidogenesis pathway, cleavage of transmembrane residue of APP by β-site amyloid precursor protein cleaving enzyme 1 (BACE-1), the main β-secretase enzyme, contributes to release β-stubs. In addition, cleavage of APP by BACE-1 leads to liberate soluble N-terminus of APP and a membrane bound C-terminal fragment (C99). At the second step, C99 fragment is cleaved by γ-secretase which contributes to Aβ release into the extracellular space [35]. The other pathway of APP processing, known as non-Amyloidogenesis pathway, is initiated by α-secretase activity. The effect of α-secretase on APP contributes to generate and release soluble APP-α (sAPPα), other APP ectodomain variant known as a neuroprotective and neurotrophic factor [35, 36]. Additionally, several roles have been noted for sAPPα, including modulation of basal synaptic transmission likely via γ-aminobutyric acid type B (GABAB) receptor subunit 1a [37]. However, explaining these two pathways can theoretically help to present therapeutic goals, although in practice more studies are required to prove this claim. In this regard, it has been proven that suppression of Amyloidogenesis pathway through suppression of BACE-1 and γ-secretase exhibits protective effects in different models of AD [38]. Similarly, inducing the non-Amyloidogenesis pathway via increasing the expression or activity of α-secretase leads to reduce Aβ production and accumulation [39]. As described, ADAM10 is one of the main α-secretases, and it has been presented as a suitable therapeutic target to modulate Aβ production.

### ADAM10 and TREM2 in Alzheimer’s disease

Triggering receptor expressed on myeloid cells-2 (TREM2) is a receptor located on cell surface and consists of a V-immunoglobulin extra-cellular domain and cytoplasmic tail [40]. TREM2 is mainly expressed in myeloid cells including granulocytes, dendritic cells, tissue-specific macrophages like osteoclasts, alveolar macrophages and Kuppfer cells [41]. In CNS, TREM2 is expressed in microglia cells and plays a great part in regulation of their activity [42]. Physiologically, TREM 2 is involved in regulation of phagocytosis, cell proliferation, and inflammation via its effect on downstream targets including the PI3K/AKT and ERK1/2 signaling pathways [41]. However, increased expression of TREM2 has been detected in different pathologies, such as Parkinson’s disease, traumatic brain injury, and AD [41] indicating its probable role in pathophysiology of these diseases. In AD, the most important role noted for TREM2 is its interactions with Aβ plaques and regulation of neuroinflammation, in a way that TREM2 is involved in microglia recruitment to Aβ plaques [42, 43]. Activation of mentioned intracellular pathways followed by TREM2-Aβ axis contributes to enhance Aβ clearance and induce inflammatory responses [35]. In addition, there is a soluble form of TREM2 (sTREM2) which is generated followed by the effect of α-secretase on extracellular domain of TREM2 [44]. The sTREM2 is known as a neuroprotective factor due to its role in enhancement of Aβ plaque degradation [45]. However, it seems that the most important α-secretase involved in TREM2 shedding and generation of sTREM2 is ADAM10. In this regard, it can be referred to a study indicating ADAM10 role in TREM2 shedding [46]. In this study it was shown that TREM2 shedding at the H157-S158 bond and generation of sTREM2 inhibited by metalloprotease inhibitors, G1254023X and siRNA targeting ADAM10, but not matrix metalloproteinases 2/9, and ADAM17 siRNA. This process can be expressed as a therapeutic target to increase sTREM2 levels through ADAM10 activation, and further studies in this regard can be constructive.

### ADAM10 and microRNAs in Alzheimer’s disease

microRNAs (miRs) are a group of non-coding RNA molecules involved in regulation of the expression of proteins through binding to 3’UTR of their mRNA [47]. In addition to their physiologic roles, it is clearly understood that aberrant expression of miRs plays a great part in different pathologies, such as cancers and neurologic disorders [48]. In recent years, numerous studies indicate that miRs are involved in AD pathophysiology, as their up- or down-regulation has been detected in these patients. In addition, pre-clinical studies demonstrate that miRs regulate different processes in AD, the most important of which are Aβ production (reviewed at [49]). In this regard, miRs have been shown to regulate the activity of α-secretase, β-secretase, and γ-secretase. In a computational study, it was reported that miR-103, miR-1306, and miR-107, which their aberrant expression has been detected in AD patients, may affect the expression of ADAM10 to regulate APP processing [50]. miR-221, a downregulated miR in AD, has been shown to reduce the expression of ADAM10 in SH-SY5Y cells [51]. In another study, it has been indicated that miR-144 and miR-451 regulate the expression of ADAM10 in HeLa and human neuroblastoma SH-SY5Y cells [52].

Collectively, these studies clarify the essential role of miRs to regulate ADAM10 expression and activity. However, more studies are required to present other miRs involved in this process and introduce them as therapeutic options in AD.

### Regulation of ADAM10 expression and activity by intracellular pathways in Alzheimer’s disease

The expression of ADAM10 is regulated at different stages of transcription (i.e. retinoic acid, sirtuins, SOX-2, and PAX-2), translation, and post-translation levels (reviewed at [53]). However, there are several intracellular pathways with altered activity in AD which their relationship with ADAM10 has been less analyzed. Extracellular signal‑regulated protein kinase (ERK)1/2 is one of the main intracellular pathways involved in regulation of different aspects of cell life, including cell proliferation and protein synthesis [54]. However, this pathway has been shown to be disrupted in patients with AD and play a role in regulation of Aβ production, tau phosphorylation, and neuroinflammation [35]. Regarding the effect of ERK1/2 on ADAM10, it has been demonstrated that ERK1/2 induces ADAM10 expression through induction of cAMP-response element binding protein (CREB) activity leading to enhance APP processing and sAPPα production [55]. Additionally, S100A7, a novel AD biomarker, has been shown to promote non-Amyloidogenesis pathway through induction of ADAM10 by mediating of ERK1/2 [56].

Phosphatidylinositol 3 kinase (PI3K)/AKT signaling pathway is the other main intracellular pathway with altered activity in AD [57]. In this regard, it has been indicated that activation of estrogen receptors contributes to promote non-Amyloidogenic processing of APP through activation of the PI3K/AKT pathway leading to enhance ADAM10 activity [58].

**Fig. 1:**
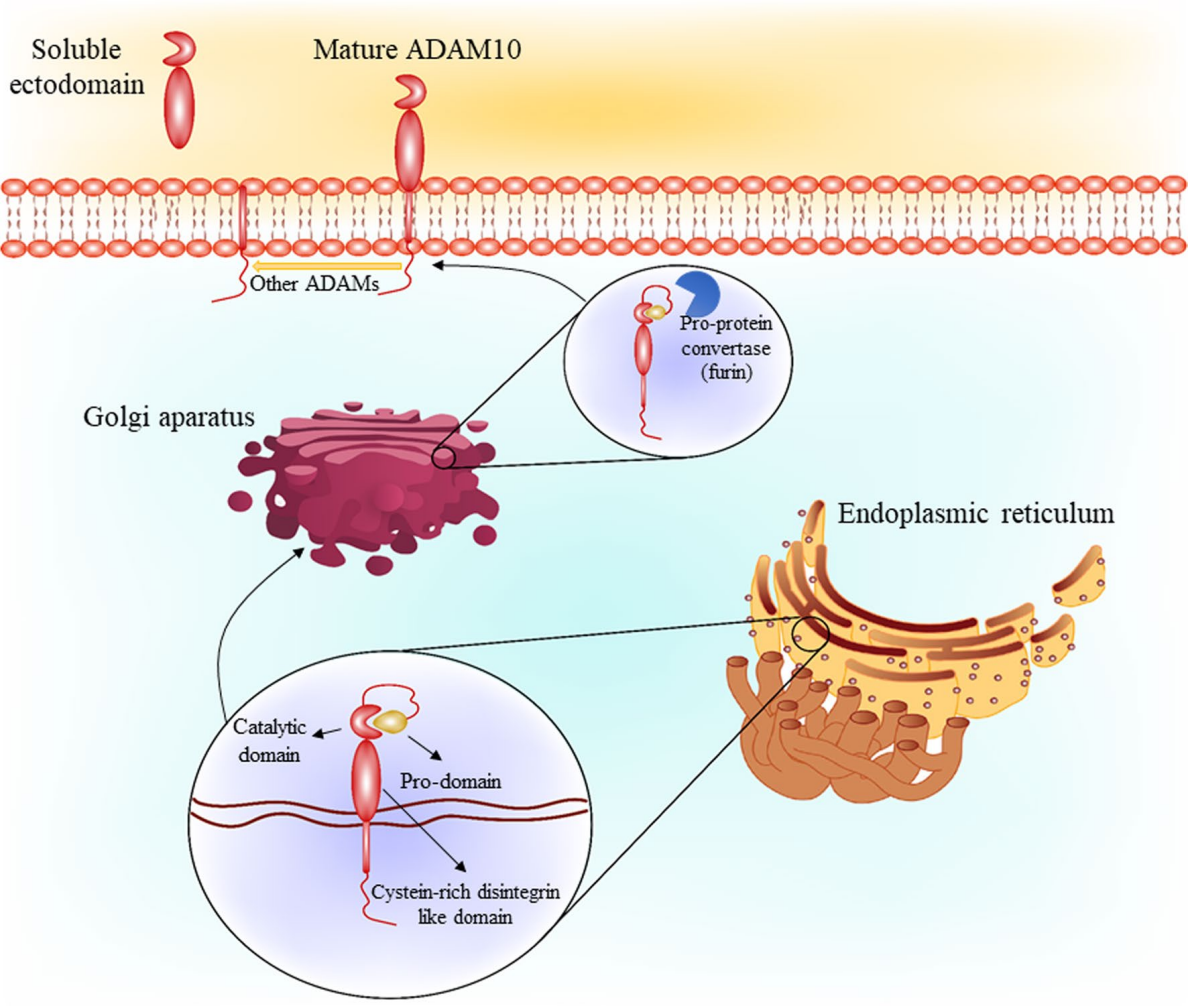
ADAM10 synthesis and maturation in the cell. ADAM10 in synthesized in endoplasmic reticulum, containing pro-domain, catalytic and cysteine-rich disintegrin like domains. Golgi is involved in ADAM10 maturation, where pro-domain is dissociated by pro-protein convertases such as furin. On the other hand, other ADAMs can affect mature ADAM10 to form a soluble ectodomain

**Fig. 2:**
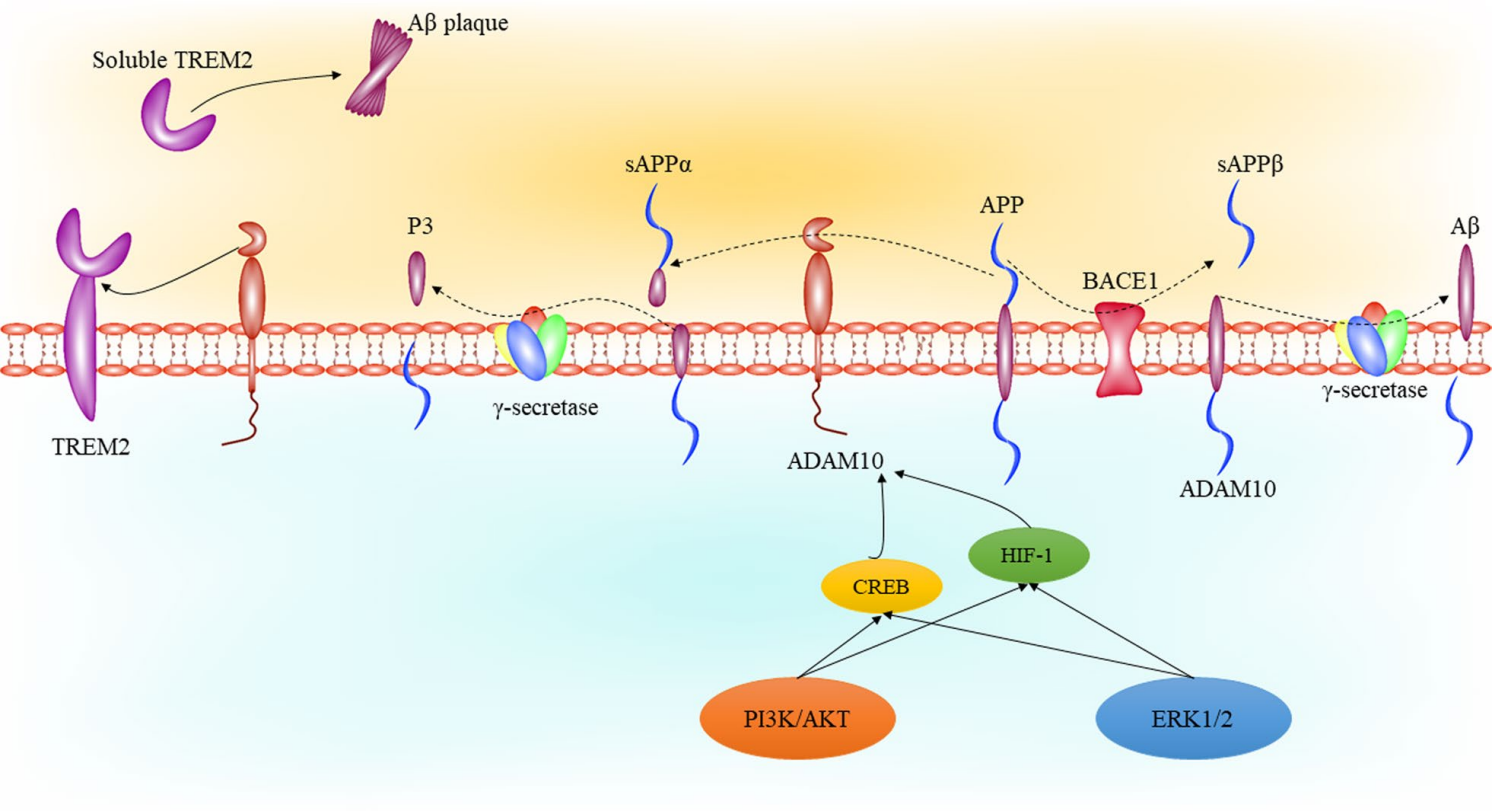
The amyloidogenic and non-amyloidogenic pathways of APP processing. In amyloidogenic pathway, BACE1 affects APP leading to release sAPPβ. γ-secretase is the other protease involved in amyloidogenic pathway, which its activity contributes to Aβ formation. In the non-amyloidogenic pathway, at the first step, ADAM10 cleaves APP leading to release sAPPα. At the second step, γ-secretase activity leads to P3 fragment formation and release. On the other hand, ADAM10 cleaves TREM2 leading to release soluble TREM2, which binds to Aβ plaque and induces its clearance. The PI3K/AKT and ERK1/2 pathways are involved in regulation of ADAM10 expression. This effect can be mediated by CREB and HIF-1. Aβ: amyloid β; ADAM10: a disintegrin and metalloproteinase domain-containing protein 10; APP: amyloid precursor protein; BACE1: beta-site amyloid precursor protein cleaving enzyme-1; CREB: cAMP response element-binding protein; ERK1/2: extracellular signal‑regulated protein kinase 1/2; HIF-1: hypoxia inducible factor 1; PI3K: phosphatidylinositol 3 kinase; TREM2: triggering receptor expressed on myeloid cells 2

Although the role of these two pathways in the regulation of ADAM10 activity is inevitable, the exact mechanism of this regulation in AD is still unknown. However, in non-AD models it has been noted that PI3K/AKT and ERK1/2 signaling pathways regulate several factors involved in regulation of ADAM10 expression, such as Y sex determination region (SRY)-box 2 (SOX-2) [59, 60]. However, more studies are required to determine the exact mechanism of the involvement of the PI3K/AKT and ERK1/2 pathways in regulation of ADAM10 expression and investigate them as a therapeutic target in AD (Figs. 1, 2).

### ADAM10 and synaptic plasticity in Alzheimer’s disease

Different experiences, whether they be stressful event, learning in a classroom, or using of a psychoactive substance, influence the brain through modifying the activity of specific neural circuitry. Synaptic plasticity is an experience-dependent change in neuronal connection strength that provides the basis for most learning and memory models [61]. This type of cellular learning consists of specific stimulation protocols generating a long-term synapse strengthening, known as long-term potentiation (LTP), or a weakening of the said long-term synapses, known as long-term depression (LTD). Although AD is known to be associated with loss of neurons in different regions of the brain, the hypothesis indicating the alteration in the molecular mechanisms of synaptic plasticity underlying this imbalance is widely accepted [62]. However, the association between various molecular aspects of AD and synaptic plasticity has been investigated in different studies.

**Fig. 3:**
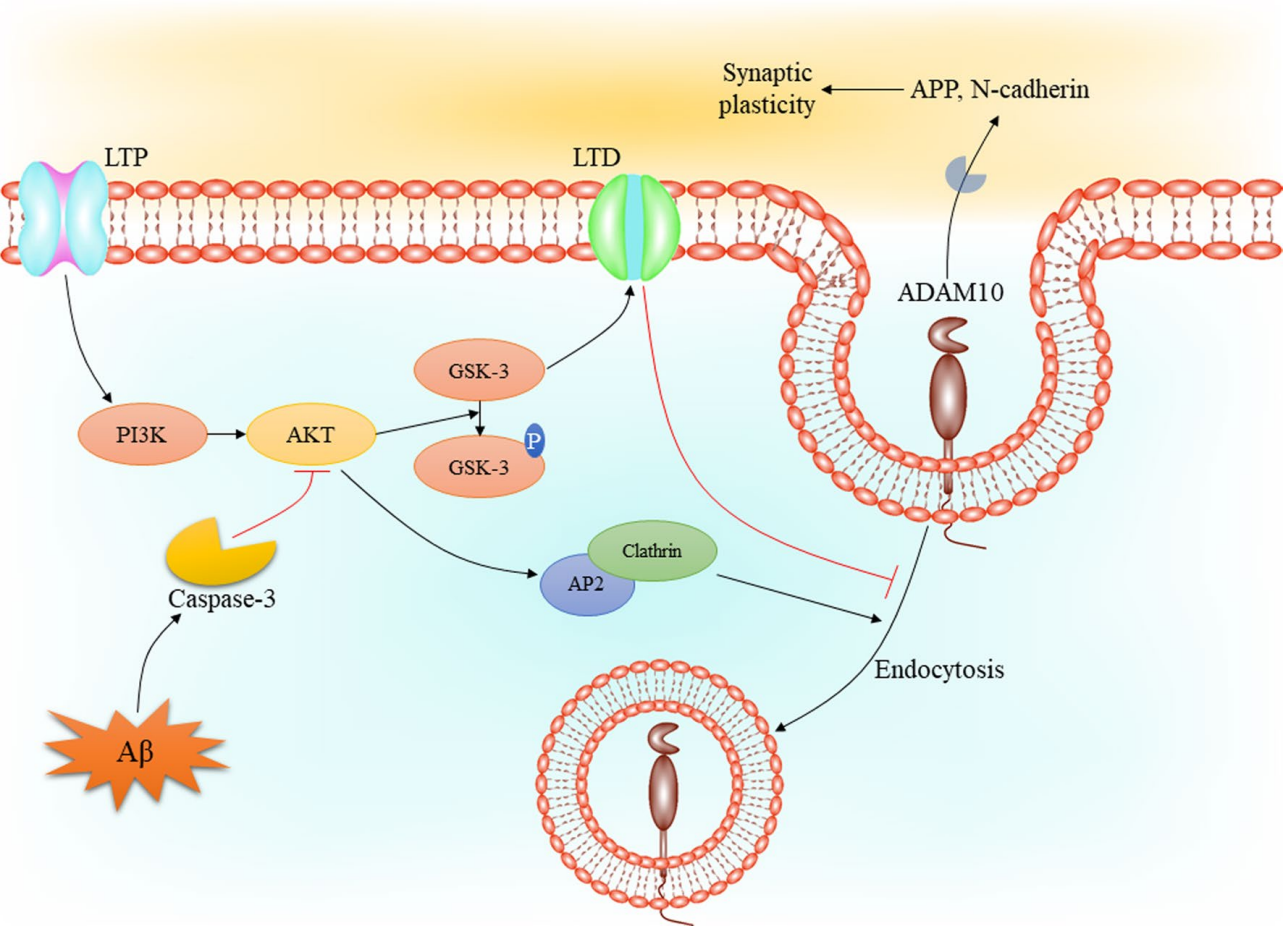
The cross-talk between intracellular pathway involved in synaptic plasticity and ADAM10 availability. LTP induces the activation of the PI3K/AKT pathway which regulates GSK-3 activity negatively. This process contributes to inhibit LTD followed by GSK-3 inhibition. On the other hand, activated AKT induces clathrin-mediated ADAM10 endocytosis via its role in regulation of AP2. Aβ can inhibit mentioned pathways through activation of caspase-3. Aβ: amyloid β; ADAM10: a disintegrin and metalloproteinase domain-containing protein 10; APP: amyloid precursor protein; GSK-3: glycogen synthase kinase; LTD: long-term depression; LTP: long-term potentiation; PI3K: Phosphatidylinositide 3-kinase;

During AD progression, the efficiency of synapses is decreased followed by a significant decrement in synaptic vesicles. In this regard, progressive changes in gene expression in CA1 region of MCI and AD brains occurs in a way that a significant decrement in different steps of synaptic function-related proteins has been detected in these patients [63]. In a closer inspection, reduced expression of synaptophysin and synaptogyrin (presynaptic vesicle trafficking proteins) syntaxin 1and synaptotagmin (vesicle coupling/fusion/release proteins), and PSD-95 (postsynaptic function regulators) has been detected in CA1 of subjects with MCI and AD [63]. In the case of Aβ, it has been observed that Aβ oligomers alter molecular pathways involved in LTP which initiate LTP decline and LTD increase in hippocampus slices [64]. Impaired learning and memory followed by Aβ injection into the brain of mice may prove the effect of Aβ on synapse plasticity [65]. These results may provide an insight into the role of ADAM10 in synaptic plasticity via modulation of Aβ production, in a way that reduced ADAM10 activity in AD brains can alter synaptic plasticity by mediating of Aβ accumulation. On the other hand, the interaction between synaptic plasticity-related processes and ADAM10 activity has been shown in several studies. In this regard, it has been reported that LTD differentially regulates the synaptic availability and activity of ADAM10 via promoting its endocytosis [66]. Additionally, in this study it was shown that synapse-associated protein 97 (SAP97) is required for LTD-induced ADAM10 trafficking. In addition to SAP97, LTP has been shown to trigger ADAM10 association to clathrin adaptor AP2, as shown to be increased in AD brains, leading to induce its endocytosis [66, 67]. Interestingly, it has been reported that Aβ oligomers-induced aberrant plasticity process can reduce Aβ generation via modulation of ADAM10 synaptic availability [68]. These results have led to introduce ADAM10 endocytosis as a suitable target for therapy in AD. In this case, it has been elucidated that treatment of APP/PS1 mice with a cell-permeable peptide (PEP3), which interferes with ADAM10 endocytosis, contributes to upregulate the postsynaptic localization and activity of ADAM10 and eventually, promote synaptic plasticity and improve cognitive deficit [69]. However, there is a long way to use these agents in clinical trials because of their non-specific activity and probable side effects. In this regard, using of agents which modulate several intracellular pathways may be considered to modulate the endocytosis of ADAM10. For instance, the PI3K/AKT signaling pathway has been shown to inhibit clathrin-mediated endocytosis via modulation of AP2 activity [70, 71]. On the other hand, it has been reported that inhibition of the PI3K/AKT signaling pathway by Aβ plaques contributes to downregulate synaptic related proteins, such as synaptophysin and post-synaptic density-95, which eventually may influence ADAM10 synaptic availability [72]. Also, it has been shown that activation of the PI3K/AKT pathway promotes dendrite branch density and increase synaptic protein expression leading to increased levels of ADAM10 in AD mice [73]. In a mechanistic inspection, it can be said that changes to synaptic plasticity may influence intracellular pathways, mainly the PI3K/AKT pathway [74], which is known as a regulator for ADAM10 expression and endocytosis. This process may be mediated by GSK-3, as it has been demonstrated that LTP-induced PI3K/AKT/GSK-3 regulates LTD in CA1 pyramidal neurons negatively [75, 76]. Therefore, it can be concluded that activation of LTP inhibits LTD along with activation of the PI3K/AKT pathway leading to induce clathrin-mediated endocytosis of ADAM10. On the other hand, caspase-3, which plays a crucial role in the pathophysiology of AD [77], has been shown to be activated by Aβ oligomers, which in turn cleaves AKT, activates LTD [78], and possibly inhibits endocytosis of ADAM10 (Fig. 3). Regardless the role of synaptic plasticity in regulation of ADAM10 availability, it has been elucidated that function of ADAM10 regulates the synaptic plasticity, mainly via cleavage of several factors such as APP, neuroligin 1, and N-cadherin [79].

### Pharmacologic modulation of ADAM10 in Alzheimer’s disease

According to the important role of ADAM10 in the processing of APP, it is clearly understood that inducing its expression or activity in AD exhibits neuroprotective effects. Numerous studies investigate the effect of different drugs and natural compounds on AD by mediating of ADAM10.

### FDA-approved drugs for Alzheimer’s disease

Although AD is known as one of the main challenges for health systems worldwide, just two classes of drugs are approved for using in AD patients, namely N-methyl d-aspartate (NMDA) antagonists and cholinesterase enzyme inhibitors (naturally derived, synthetic hybrid analogues). Cholinesterase enzyme inhibitors are used in AD based on the cholinergic hypothesis which proposes the reduction of acetylcholine (ACh) biosynthesis in AD. NMDA receptor antagonists, the other group of FDA-approved drugs for AD, are used due to overactivation of NMDA receptors in the brains of AD patients which results in an elevation in Ca^2+^ influx to the neurons and subsequently induction of oxidative stress and neuronal apoptosis [80, 81]. However, the neuroprotective effects of mentioned drugs on other pathologic changes in AD has been shown in several studies. In this regard, it has been reported that memantine, a NMDA receptor antagonist, induces the expression and activity of ADAM10 and reduces Aβ oligomers formation in 3xTg-AD mice leading to improve cognitive function [82]. Additionally, it has been observed that rivastigmine, a cholinesterase enzyme inhibitor, up-regulates the ADAM10 levels leading to increase sAPPα generation in 3 × Tg mice [83]. Regarding the other cholinesterase enzyme inhibitor, donepezil, it has been elucidated that treatment of SH-SY5Y cells by donepezil increases sAPPα formation via induction of ADAM10 activity [84].

### Melatonin

Melatonin is an endogenous hormone responsible for regulation of circadian rhythm, free radicals, and neuroprotection [85]. In addition, melatonin is also prescribed in patients with sleep disorders [86]. However, the association between melatonin and ADAM10 activity is very interesting. It is clearly understood that melatonin is an inducer for ADAM10 transcription through direct effects on the promoter regions 2304 and 1193, subsequently, increase ADAM10 expression [87, 88]. Decreased levels of melatonin in AD patients has been introduced as a mechanism for decreased ADAM10 expression and Aβ accumulation in these patients [89, 90]. However, the mechanism of melatonin-induced ADAM10 expression has been well-studied. In this regard, it has been shown that melatonin induces ERK1/2 phosphorylation via binding to melatonin receptor, and increases ADAM10 expression leading to up-regulate non-Amyloidogenic pathway of APP processing [87]. Also, melatonin has been indicated to induce ADAM10 expression and suppress BACE1 expression through activation of melatonin G protein-coupled receptors in human neuronal SH-SY5Y cells [91]. It has been demonstrated that melatonin increases ADAM10 expression in the hippocampus of aged mice through upregulation of sirtuin1, one of the main regulators of ADAM10 transcription [92].

### Statins

Statins are a group of lipid-lowering agents. They suppress conversion of 3-hydroxy-3-methylglutaryl coenzyme (HMG-CoA) to through inhibition of HMG-CoA reductase [93]. In addition to effectiveness of statins in regulation of lipid levels, they are known due to their pleiotropic effects, including immunomodulatory, anti-oxidant, and anti-tumor properties [94]. Also, statins exert neuroprotective effects in different neurologic pathologies, such as neurodegenerative diseases [95]. In AD, these drugs affect different pathologic processes, especially Amyloidogenesis and APP processing [96]. In this regard, it has been shown that statins increase APP processing leading to generate sAPPα in N2a mouse neuroblastoma cells through an isoprenoid-mediated mechanism, and possibly ADAM10 induction [97]. In addition, it has been demonstrated that atorvastatin increases α-secretase activity and stimulates sAPPα production in N2a cells through an ERK-independent mechanism [98].

### Natural compounds

In recent years, numerous studies focused on the protective effects of different natural compounds on AD. Although these agents are best known because of their anti-inflammatory and antioxidant properties, they can have far-reaching effects on other aspects of AD pathophysiology. One of the most important effects of natural compounds on AD is their role in modulating Aβ production, which is mediated by their effect on the expression of factors involved in its production, such as BACE1, γ-secretase, and α-secretase. However, the association between different natural compounds and ADAM10 expression and activity in models of AD summarized in Table 2.

**Table 2:** Natural compounds-induced ADAM10 modulation in AD

Natural compound	Source	Model	Effect	Result	References
Curcumin	Curcuma longa	HEK293 cells overexpressing ADAM10	Increase ADAM10 expression	Increase sAPPα production and exert neuroprotective effects	[107]
Icariin	Epimedium brevicornum	Rat model of permanent occlusion of bilateral common carotid arteries	Reduce BACE1 and increase ADAM10 expression	Decrease in the level of insoluble Aβ fragments	[108]
Astaxanthin	Euphausia pacifica	3xTg AD mice	Induce ADAM10 expression	Up-regulation of non-Amyloidogenesis pathway	[109]
Epigallocatechin-3-gallate	Green tea	SweAPP N2a cells	Increase active form of ADAM10 (∼ 60 kDa mature form)	Increase sAPPα production and exert neuroprotective effects	[110]
		SweAPP N2a cells	Activation of ADAM10 through the PI3K/AKT pathway activation	Increase sAPPα production and exert neuroprotective effects	[111]
Resveratrol	Polygonum cuspidatum	CHO-APPswe cells	Augment altered expression and subcellular localization of ADAM10 induced by high cholestrol	Up-regulation of non-Amyloidogenesis pathway	[112]
Bilobalide	Ginkgo biloba	Differentiated SH-SY5Y cell	Induction of ADAM10 through the PI3K/AKT pathway activation	Increase sAPPα production and exert neuroprotective effects	[113]
Quercetin	Hypericum perforatum	Aluminum chloride -induced AD rat model	Up-regulation of ADAM10	Inhibit Aβ aggregation	[114]
Ligustilide	Angelica sinesis	APP/PS1 mice	Up-regulation of ADAM10	Inhibit Aβ aggregation	[115]
Cryptotanshinone	Salvia miltiorrhiza	N2a-SwedAβPP cells	Activation and translocation of ADAM10 and PKC-alpha	Up-regulation of non-Amyloidogenesis pathway	[116]
Berberine	Berberis vulgaris	APP/PS1 transgenic mice	Increase ADAM10 expression	Reduce Aβ deposition and improve memory function	[117]

## Conclusion

It is clearly understood that ADAM10 has a protective effect against AD progression due to its role in processing of APP in non-amyloidogenic pathway. Pharmacologic evidence also suggests its potential as a therapeutic target in AD. In addition, alterations in ADAM10 expression and activity in different samples from AD patients can be encouraging to introduce it as a therapeutic option in AD. However, there are different natural compounds, with low side effects, which can be used in clinical trials due to their effects on ADAM10.

## Data Availability

Not applicable.
